# Rethinking the Negativity Bias

**DOI:** 10.1007/s13164-018-0382-7

**Published:** 2018-02-05

**Authors:** Jennifer Corns

**Affiliations:** Philosophy, School of Humanities, University of Glasgow, 67-69 Oakfield Avenue, Glasgow, G12 8QQ UK

## Abstract

The negativity bias is a broad psychological principle according to which the negative is more causally efficacious than the positive. Bad, as it is often put, is stronger than good. The principle is widely accepted and often serves as a constraint in affective science. If true, it has significant implications for everyday life and philosophical inquiry. In this article, I submit the negativity bias to its first dose of philosophical scrutiny and argue that it should be rejected. I conclude by offering some alternative hedonic hypotheses that survive the offered arguments and may prove fruitful.

## Introduction

Consider the following cases:

- In a single day you receive both a rejection and an acceptance from comparably prestigious journals for two articles in which you are equally invested. The day is a difficult one: the deflation you feel from the rejection overwhelms the elation you feel from the acceptance.
- During an evening at the pub, you are introduced to a friend of a friend who is gregarious and seemingly intelligent. You strike up a conversation and discover that your new acquaintance is a social worker who volunteers at the local food bank and you are impressed with their kindness. After a pint, they make an offensive, racist comment. You disentangle yourself from the conversation and do not speak to them again.
- You are enjoying a sunny, Autumnal day off with a beloved. Strolling through the park after a relaxing morning, you encounter a colleague who nonetheless—you are sure they see you wave—snubs you. The slight nags at your mind and, despite engaging in many of your favourite recuperative activities, you cannot dismiss it.

In these cases, the bad happenings are apparently more efficacious than some seemingly equivalent or greater goods. Moreover, cases like these apparently proliferate. No matter how many nice things you may have said to me in the past, one serious insult can ruin our relationship. A single lie or infidelity can destroy an otherwise happy marriage. Negative occurrences in an affluent childhood can result in a turbulent life. A delicious meal can be ruined by the touch of an insect, and an upset stomach following the meal can forever preclude enjoyment of the consumed food type. The negative, then, is of seemingly greater importance to us: it consumes our attention, informs our opinions, and generally affects us disproportionality to the positive.

Consideration of cases like these has led to expansive testing of the seemingly asymmetrical causal profiles of the negative and the positive. In search of a unifying principle, many empirical studies have now been done comparing the effects of some good to the effects of some corresponding bad. As discussed below, this research encompasses comparing a wide range of causes across a wide range of effects. Studied causes include monetary rewards and losses, positive and negative traits, pleasant and unpleasant foods, and so on. The considered effects of these compared causes include health outcomes, retention in memory, facilitation of learning, longevity of relationships, and much more.

In the wake of the results of this extensive research, it is now widely accepted among psychologists and neuroscientists that there is a general psychological principle, holding for humans and perhaps all mammals, that the negative is more causally potent than the positive. Further elaborated below, this principle is called *the negativity bias*. As it is often more colloquially stated: bad is stronger than good. The negativity bias currently serves as a common starting place for, and a constraint upon, further inquiry in affective science. Its import, however, is not limited to these empirical inquiries.

Potential practical implications abound. In education, application of the principle may involve preferring punishments to praise for facilitating learning. Though ethical constraints would need to be respected, negative reinforcements would be more efficacious over their positive counterparts. Similarly in the workplace: the knowledge that disincentives are stronger than incentives and criticism stronger than praise could be exploited within ethical limits. Likewise in politics: the principle implies that smear campaigns will indeed be more effective than positive campaigns and cautions that one misjudged act may ruin otherwise pristine reputations. Recognizing the principle may also prompt attempts to counterbalance its effects, promising benefits for conflict resolution. Finding myself angry at someone, if I recognize that I am likely weighing the negative stronger than the positive, I may consciously focus on the positive in an attempt to counterbalance my judgement of the offender’s wrongdoing. Though some may already accept some of these implications, the negativity bias holds out the hope of a unifying explanation that may receive appeal for clarification or justification, and spur yet further, novel applications.

Similarly, the negativity bias has broad implications for many philosophical inquiries. A few of these have recently received initial attention by Adam Shriver (2014) and Olivier Massin (2014). Shriver (2014) has argued that pain and pleasure make asymmetrical contributions to well-being in virtue of an intrinsic connection between motivation and negative affect which fails to hold for positive affect. The negativity bias—and its wide acceptance among affective scientists—is explicitly a key plank in his argument, offered as evidence that the causal profiles of the negative and the positive are importantly divergent. Massin (2014) acknowledges the negativity bias, but nonetheless argues for a “moderate optimism.” On this view, though bad may be stronger than good, in many domains there may be *more* good than bad, such that the implications of the otherwise undisputed negativity bias are blunted.

The few philosophical implications discussed so far are the tip of the iceberg. As Shriver points out, since the parity and symmetry of the negative and the positive have generally been taken for granted, the negativity bias has profound implications for ethics. Claims about the negative should no longer be taken to automatically entail corresponding claims about the positive, or vice versa. If the negativity bias is accepted, the many arguments relying on this faulty entailment need re-evaluating. The supposed greater causal power of the negative over the positive—of pain over pleasure, bad over good—thus has unexplored significant ramifications for ethics and moral psychology. In the philosophy of mind, inquiries into the nature of affective states—from bodily sensations like pain, to complex emotional states like jealousy—likewise take symmetry and parity for granted. Though these affective states are of increasing interest to philosophers, current theorizing continues to assume that what is true for either type of affect is true for both. For empirically inclined and informed philosophers of mind, science, and cognitive science, moreover, evaluation of the principle may be of interest in its own right.

The negativity bias is thus an intuitively plausibly principle that is widely accepted within affective science which has significant implications for both everyday life and philosophical inquiry. Though philosophers have yet to pay it much attention, the time is ripe for so doing. If the negativity bias is true, it is time to adjust our philosophical theories accordingly, and acknowledge the principle—if only to attempt to counterbalance it—in our daily lives. Given its current influence in affective science and encroachment into philosophy, if it should instead be rejected, it is time to dispute it.

In what follows, I submit the negativity bias—as currently proffered and accepted in contemporary psychology—to its first dose of substantial philosophical scrutiny.^1^ I argue that it is problematically formulated and that its supposed empirical support is credibly explained by alternative hypotheses. In the following section, I focus on the formulation of the hypothesis, and in section three, I focus on alternative explanations for some of the results of scientific inquiries offered in supposed support of the hypothesis. In section four, I conclude by offering some alternative hypotheses for subsequent investigation that may prove more fruitful. If the following arguments are sound, then despite currently receiving unquestioning support across a range of disciplines, the negativity bias should be rethought.

Space clearly precludes discussion of all (actual or possible) formulations of the negativity bias or all the research which has or may be offered in its support. Accordingly, the following discussion is centred on Baumeister et al. (2001). A word should be offered in defence of this methodology.

In both psychology and neuroscience, Baumeister et al. (2001) are taken to have offered the cannonical formulation of the negativity bias, along with a profundity of supposed evidence. Their review continues to be the paper most often cited in current work—work which cites Baumeister et al. as having established the bias.^2^ As their landmark piece serves as the as yet unquestioned foundation, it is particularly worthy of scrutiny; even if the following criticisms applied only to this core piece, they would remain worth offering publicly. The contained criticisms, however, in fact encompass the literature. The ambiguities and contradictions argued to undermine the foundation of the negativity bias thus threaten the profundity of work that has been built upon it.

## The Hypothesis

Baumeister et al.’s (2001) claim is that bad is stronger than good.^3^ In one summary of this titular hypothesis, they write “When equal measures of good and bad are present… the psychological effects of bad ones [events] outweigh those of the good ones [events]” (323). Variations on this claim have come to be known as the *negativity effect* or the *negativity bias* and, as introductorily noted, it is now widely accepted.^4^ Unfortunately, the hypothesis is never adequately clarified, creating a host of hitherto unrecognized problems.

The authors seem to assume that their key terms—‘bad’, ‘good’, and ‘strong’—require no clear definition, and indeed that none can be given, because they are “universal and fundamental”. Accordingly, in their introduction they nevertheless attempt to assuage worries about defining their key terms by writing (p.325):

Definition implies rendering one concept in terms of others, and the most fundamental ones therefore will resist satisfactory definition. *Good*, *bad,* and *strength* are among the most universal and fundamental terms… and it could be argued that they refer to concepts that are understood even by creatures with minimal linguistic capacity (such as small children and even animals). By good we understand desirable, beneficial, or pleasant outcomes including states or consequences. Bad is the opposite: undesirable, harmful, or unpleasant. Strength refers to causal impact. To say that bad is stronger than good is thus to say that bad things will produce larger, more consistent, more multifaceted, or more lasting effects than good things.

For clarifying their hypothesis, however, this will not do. First, ‘good’, ‘bad’, and ‘strong’ remain unclear in ways that matter for evaluating the hypothesis. Second, the problems arising from this unclarity are exacerbated by the lack of clarity concerning the subjects to which the key terms are being applied, i.e. what the hypothesis is supposed to be *about*. Consider each in turn.

### Predicates

‘Good’, ‘bad’, and ‘strong’ are all unclear in ways that matter for evaluating the hypothesis.

First consider ‘strength.’ In the passage quoted above, the authors say that “strength refers to causal impact.” What kind of causal impact and how is its strength to be evaluated? They seem to acknowledge that further clarification is required by initially specifying a stronger effect as one that is “larger, more consistent, more multifaceted, or more lasting” than another.

Unfortunately, however, these are distinct hypotheses. More multifaceted and more lasting, for instance, are distinct and dissociable measures: some effects may last longer while being less multifaceted than brief others, and conversely some effects may last a short while being highly multifaceted. The same problematic dissociations arise for consistency. There is no reason to think these three specifications of strength are measures of the same thing and even brief consideration suggests that they are not. One may, of course, operationalize ‘strength’ in a given context in any way one likes—but *consistency* across the supposed evidence offered in support of claims about strength is required.

The problematic conflation across notions of strength is seen in the supposed evidence offered by the authors throughout the text, as the meaning of ‘strength’ becomes increasingly stretched. A wide range of measures are blithely offered. In addition to the initial three specifications, a thing is taken by Baumeister et al. to be stronger than another as a matter of the degree to which: one is motivated by it (p. 351); it produces emotion (p.328); it affects adjustment measures (p. 328); it predicts marital longevity (328); it is “pronounced” (p.330); people agree about its application (p.330); it influences opinion (p.331); it is “important” (p. 332); it is avoided by a wide range of techniques (p. 332); it takes time to process (p. 334); the elaboration with which it is processed (p. 340); one makes decisions concerning it (p. 334); it facilitates learning about other things (p. 335); it is itself learned about (p. 336); it causes a “response in the brain” (336); it is remembered (337); and it predicts distress (340). This list is not nearly exhaustive, but includes more than enough to be perplexing.^5^

Many of these senses of ‘strength’ are regrettably vague, but insofar as we can specify them, they are again distinct and dissociable. Counterexamples come easy for almost any of the above two criteria. I may use only one technique to deal with my fear of spiders (e.g. try to get away), but that fear may nonetheless be a good predictor of my distress and a poor predictor of my marital longevity. In considering the multitudinous criteria, in Baumeister et al. alone and much less beyond, it begins to looks as if ‘strength’ is allowed to mean almost anything that we can measure. But if that is right, then it is not clear what the hypothesis amounts to. Again, the problem is not that we need a once-and-for-all operationalization of strength for all hypotheses that we might want to test, but to know whether some good X is stronger than some bad Y, we’ve got to know what ‘stronger’ means as it occurs in the negativity bias. Without this, we can neither confirm nor disconfirm the hypothesis. Worse still, with conflicting measures we could both confirm and disconfirm the hypothesis with the same data. The lack of clarity concerning ‘strength’ is thus deeply problematic.

The laxity concerning ‘strength’ and how strength is measured is what appears to lead contradictory results to be offered in supposed support of the hypothesis. Consider just one example of this from Baumeister et al..^6^ On the one hand, the authors claim that bad information (most perspicuously: information about something the receiver takes to be bad) and bad moods take longer to process and involve further cognitive elaboration, and they offer results in support of this claim (e.g. p. 334). *Increases* in response time, cognitive processing, and elaborated responses are thus all taken as measures of strength. On the other hand, however, the authors claim that negative information is processed faster because it is more important and they offer results in support of this claim (e.g. p. 346). *Decreases* in response time, cognitive processing, and elaborated responses are measures of strength. Because ‘strength’ is unclear, it is understood in opposite ways, and conflicting results are both taken to provide support.

More generally then, without restrictions on what ‘strength’ means and how it is supposed to be measured, any measurable asymmetries will seem to both confirm and disconfirm the hypothesis. Any difference between the effects of some bad X and good Y, that is, can be *interpreted* as evidencing either strength or a lack of strength. The authors, of course, opt to interpret all of these results as evidence of strength—and the negativity bias literature, built upon this foundation, has followed suit. You remember X longer? That’s taken to be evidence of X’s strength; it is more important, so you remember it for future. You remember Y longer? That is taken to be evidence of X’s strength; it is threatening, so you forget it as quickly as possible. You learn X more easily? That is taken to be evidence of X’s strength; you have evolved to pay more attention to it, facilitating learning. You learn Y more easily? That is taken to be evidence of X’s strength; it is painful to concentrate on X, so you allow yourself to become easily distracted. Clarification restricting the measures for strength is needed before any (subset) of these results can be legitimately accepted as evidence.

The lack of clarity concerning ‘good’ and ‘bad’ only compounds these problems. Baumeister et al. claim that “‘[g]ood’ and ‘bad’ are among the first words and concepts learned by children (and even by house pets), and most people can readily characterize almost any experience, emotion, or outcome as good or bad” (p. 323). The authors seem to think it is simply obvious whether an experience, an emotion, or an outcome is good or bad. This, I submit, is simply untrue.

One problem is that experiences, emotions, and outcomes are often good in some ways but bad in others. The authors claim that bad is stronger than good. Good or bad in what way? As with ‘strength,’ while we do not require that the authors settle some once-and-for-all meaning of ‘good’ and ‘bad,’ evaluation of the hypothesis does require consistency in the meaning of these terms as they there occur. Presumably, the required categorization is good or bad *all things considered*. But this all things considered evaluation is not straightforward—especially if, as it seems, the relevant all-things-considered good encompasses moral, prudential, and aesthetic goods. The brief specification the authors give of undesirable/desirable, harmful/beneficial, unpleasant/pleasant are not enough taken individually. Taken together, as any ethicist knows, these specifications will often conflict.

As a single example, imagine that you send a drafted piece of work to a respected colleague for feedback. The colleague generously takes the time to send you extensive feedback: along with identifying points that they believe that you have made well, they point out mistakes in your reasoning, grammatical infelicities, and gaps in your scholarship. Consider the experience of reading their feedback. Is this a good experience or a bad experience all things considered? Being both beneficial and unpleasant, it is hard to say. One might object that this example actually involves many experiences, emotions, and outcomes, each of which *is* obviously good or bad. In response then, consider the experience of reading one particular comment identifying a problem in your argument. None of this is to deny that we regularly make all things considered judgments. It is to deny that these are easy, universal, and require no theorizing. Most import for present purposes is that when these judgements are difficult, it is not any easier to make them by using any one of the authors’ criteria and, moreover, they conflict.

One might think hedonically complex experience like this one are relatively rare, allowing the authors to maintain that “almost any” experience, emotion, or outcome is easily categorized, but this seems simply not to be so. Hedonic complexity is commonplace. My lunch is delicious tasting, but artery clogging, and I think about my arteries as I eat. My session on the elliptical, to work off my fattening lunch, makes me feel healthy and proud, and also tired, sweaty, and involves an annoying pain in my ankle. Are these commonplace experiences good or bad? Whatever the answers, they are not obvious. Similar considerations apply to both emotions and outcomes. I am happy about something harmful, I am relieved to lose a hated job, I am remorseful for ending an unhealthy friendship: are these good or bad emotions and outcomes, all things considered? The hedonic complexity of many (if not most) experiences, outcomes, and emotions is such that it is simply not obvious whether they are good or bad. This is not to argue that there is no answer, but it is to say that the hypothesis cannot be evaluated without further clarification. And again, notice that the critique–if sound—extends beyond Baumeister et al.’s foundational work; while I am focused on the canonical text, use of ‘good’ and ‘bad’ and the range of measures taken to support their presence and degree are increasingly stretched and ambiguous the more of the literature that we consider. Adding yet *further* senses of ‘good’ and ‘bad’ exacerbates the problem.

The intended sense of ‘good’ and ‘bad’ for stating and evaluating the hypothesis is plagued by a number of further problems arising from variation. Consider that what is beneficial for one person can be harmful for another. So too, things that are pleasant for one person—to use another, conflicting criterion—can be unpleasant for another. I might also evaluate some experience type—say, a roller coaster ride or a horror movie—as good, while you evaluate it as bad. There is rampant hedonic variation and the authors give no indication of how the hypothesis is meant to apply in the face of it. Insofar as this variation is not taken into account, the applications and explanations made available by the negativity bias flounder. We need some further specifications to deal with the differences in what is good or bad—in these distinct ways—for distinct creatures and persons, and for the same person across times.

A difficulty evaluating the hypothesis that is acknowledged in the contemporary literature is that the compared things must be good or bad to the same degree. As Rozin and Royzman (2001) note (p. 300): “The logic or argument for negativity bias is complex, largely because of the difficulty of equating negative and positive events.” No one thinks that anything good to any degree is weaker than anything bad to any degree. Instead, the bad and good being compared must have the same *hedonic magnitude*. Comparing hedonic magnitudes is difficult enough when the senses of ‘good’ and ‘bad’ are clarified,^7^ but it does not seem to have been appreciated that without clarification of these terms, any attempts to engage in this difficult task remain unprincipled. With conflicting criteria for good and bad, controlling for hedonic magnitude becomes a mug’s game.^8^

Moreover and finally, the problematic laxity with which ‘strength’ is measured fatally exacerbates the problems of hedonic magnitude. As Peeters and Czapinski (1990) note (p. 34), “If the greater impact of a negative stimulus is due to the greater intensity of that stimulus, we do not have a genuine negativity effect but simply a trivial intensity effect.” But without further clarification, greater impact may *always* be interpreted as evidencing greater intensity. Because the measures of strength and hedonic magnitude are unrestricted, there is nothing to stop their conflation. Again, the problem is not the lack of a once and for all meaning of ‘good’ and ‘bad’—requiring that of affective scientists would be inappropriate. But we do require a single and consistent meaning of ‘good’, ‘bad’, and ‘strength’ as these occur in the hypothesis in order to evaluate it. The failure to consistently clarify the hypothesis’ key terms undermines legitimately interpreting any results of empirical inquiries as evidence or support for the negativity bias: any apparent difference in strength discovered might always, instead, be as legitimately taken to evidence a difference in hedonic magnitude. The hypothesis, then, could never be confirmed or disconfirmed. As such, it should be rejected as ill-formed. Unless the intended senses of ‘strength’, ‘good’ and ‘bad’ are clarified in a more restricted way, it is hard to see how this wholesale rejection of the hypothesis can be avoided. Notice that this problem is not a problem with Baumeister et al.’s formulation in particular; rather, the problem will arise insofar as the wide range of measures of ‘strength’, ‘good’, and ‘bad’ are all taken to support some unified, increasingly stretched, hypothesis.

### Subjects

Not only are the key terms of the hypothesis thus problematically unclear, but its subject matter is not adequately identified. What things of equal hedonic magnitude are being compared for strength? Candidates throughout the text include emotions, information, outcomes, interactions, personality traits, and more besides.

I think that the most charitable interpretation is to understand the authors not as confusing the many types of things they discuss, but as taking the hypothesis to hold equally well for all of them. There is good reason to think this is indeed what they mean. Baumeister et al. conclude their article by saying (p. 362):

In our review, we have found bad to be stronger than good in a disappointingly relentless pattern. We hope that this article may stimulate researchers to search for and identify exceptions…Given the large number of patterns in which bad outweighs the good, however, any reversals are likely to remain mere exceptions. The lack of exceptions suggests how basic and powerful is the greater power of bad.

They likewise tend to infer from the general claim to any particular subject, for instance: “If bad is generally stronger than good*,* then information pertaining to bad events should receive more thorough processing than information pertaining to good events” (p.340). They later note (p. 355) that they were “…unable to locate any significant spheres in which good was consistently stronger than bad.” It seems that the negativity bias is intended to hold for any types of things whose tokens may be good or bad. We do best to interpret the hypothesis as the claim that bad events, experiences, outcomes, information, and so on are *all* (respectively) stronger than good events, experiences, outcomes, information, and so on of corresponding hedonic magnitude.

One qualification, however, appears to be that the negativity bias must be some psychological phenomenon or other. And, indeed, the negativity bias has been taken to be a hypothesis that has been established as useful for explanation and prediction in psychology in particular. Thus they write (p.323) that the hypothesis “…may in fact be a general principle or law of psychological phenomenon.” This psychological qualification may be interpreted in at least two ways. First, it may mean that the bad is psychologically stronger than the good. In this case, the psychological entities to which the law applies are the *effects* of the good or bad thing. Second, it may mean that the psychologically bad is stronger than the psychologically good. In this case, the psychological entities to which the law applies are the psychological states which are themselves good or bad, and *causes* of asymmetrically strong effects. Again, the authors appear to endorse both interpretations: the bad has a stronger psychological impact than the good *and* the psychological good is stronger than the psychological bad. And again, the subsequent literature has unquestioningly followed suit.

It is important, however, to keep clear whether the subjects of the hypothesis are inputs to psychological states, e.g. events, or are instead psychological states themselves, e.g. emotions.^9^ Conflating these creates problems.

One problem is an intensification of those already seen, because the intelligible senses of ‘good’, ‘bad’ and ‘strong’ are limited by their subjects. A cup of coffee may be good, bad, or strong in different ways than a shot of whisky—and in virtue of different features. Similarly, the features in virtue of which a mental episode is good are distinct from the features in virtue of which an external event is good. The conflation of subject to which the hypothesis is supposed to apply is, I suspect, one source of the problematic lack of clarity concerning the intended predicates. Unless we are clear on the subjects, it will be hard to specify the predicates as needed to evaluate the hypothesis.

Another problem is that mental episodes, in particular the emotions, are sometimes taken to be effects by which to evaluate causes, while at other times they are taken to be causes which are to be evaluated by their effects. When they are taken as the effect of a valenced cause, they are taken to serve as a measure of the strength of the causes being evaluated. When they are taken as the valenced cause being evaluated, they are instead measured for strength by their distinct effects. So, on the one hand, when evaluating the evidence concerning the way that people react to events, Baumeister et al. take emotions to be the effect of valenced causes, summarizing (p. 328): “…most findings indicate that people react more strongly to bad events than good events. …. Bad events produce more emotion, have bigger effects on adjustment measures, and have longer lasting effects.” Later, however, the authors take the emotions themselves to be the valenced causes being evaluated, writing (331): “The prediction [of the negativity bias] is that negative affect and emotional distress will have stronger effects than positive affect and pleasant emotions...”. There is nothing illegitimate about evaluating both what causes emotions and the effects of emotions, but whether the emotions are being understood as the hedonic cause of an effect or instead as the effect of a hedonic cause matters for the different predictions and explanations the negativity bias is interpreted as offering. These remain conflated across the literature.

As an example, consider the way that Bauemiester et al. draw (2001) on Baumeister and Leary (1995) to support the negativity bias. In summarizing this support, they say (p.331)

…when Baumeister and Leary (1995) reviewed the evidence in support of a need to belong, they concluded that that need was for nonnegative interactions, rather than positive ones as they had originally theorized. The reason was that neutral interactions seemed adequate to satisfy the need to belong in many cases. This too confirms the greater power of bad: The effects of positive, good interactions were not consistently different from the effects of neutral interactions, whereas bad ones were clearly different from the neutral.

But the “neutral interactions” here are ones that seem likely to be categorized as involving neutral happenings, but non-neutral mental episodes. At least, in my own case, I would categorize many of the interactions seemingly relevant to my feeling of belonging in this way. Someone saying hello, my neighbour hanging their laundry, mail in my slot, the same man being in my corner store, the familiar smells and sights on my way to the office—these are all part of the humdrum of my life. If asked, I would categorize these as neutral events. Nonetheless, and indeed perhaps partly *because* of the humdrum neutrality of these events, they also involve positive feelings of belonging. This hedonic complexity is hidden in the conflation of events and emotions.

Note that none of this is to deny that there is an important connection between the goodness and badness of things in the world and the pleasantness and unpleasantness of one’s mental episodes. Any theory of hedonics needs, ultimately, to be complemented by a plausible theory of value. The problem is the conflation of these connected things.

This problem may seem easily avoided by determining the valence of the events by the hedonics of the states they cause. This, however, spawns other difficulties. The variation across conditions, persons, and times mentioned in the previous section would again wreak havoc. So too, as we will see in section three, many of the results which have been taken to support the negativity bias involve stimuli that are also taken to have some *independently* determined valence. These results are offered even in cases where it is known that there is a poor correlation. For one early instance, in Baeyens et al. (1990), an ingested sugary substance is taken to be a ‘good’ to be compared to an ingested non-sugary ‘bad,’ despite the fact that this particular sugary substance is reported by subjects as tasting unpleasant.

Further specification of the subject to which the hypothesis is intended to apply is thus needed. In particular, the claim that bad psychological states are stronger than good psychological states is distinct from the claim that bad inputs to psychological states are stronger than good inputs to psychological states. These hypotheses mean different things, make different predictions, and would be explained by different mechanisms. This ambiguity would remain even after ‘good’, ‘bad’, and ‘strong’ were specified—though the intelligible specification of these predicates is not independent of the needed specification of the subjects. Notice again that though I have focused discussion on Baumeister et al.’s formulation, the offered criticisms concerning ambiguity and contradictions apply across the literature and intensify when extended to it.

## The Supposed Evidence

I have argued that without further, and restrictive, clarifications on both the key terms and the intended subjects to which those terms are intended to apply, the negativity bias is unacceptable. Baumeister et al., however, have drawn extensively from the relevant empirical literature and offered a wide range of results which they interpret as being evidence in favour of their hypothesis. It was this supposed empirical basis that led Baumeister et al. to offer their canonical formulation of the negativity bias. We might charitably presume that it is likewise on the basis of empirical results, interpreted as supporting evidence, that the negativity bias has been so widely accepted across the scientific community, with further results, interpreted as further evidence, continuing to mount. If what I have argued in the previous section is correct, however, the negativity bias is ill-formed such that these empirical results could just as well be interpreted as disconfirmation as confirmation, i.e. they do not actually constitute supporting evidence for the hypothesis, since—in its current form—nothing could.

Once the negativity bias has been rejected, what should we say about those results previously interpreted to be evidence? If the arguments in the previous section are correct, then this question is analogous to what a physicist in the late nineteenth century may have asked about the supposed evidence taken to support hypotheses concerning luminiferous aether.

In this section, I offer some alternative, cross-cutting explanations for some subsets of those results which have been supposed to be evidence for the negativity bias. Though space precludes exhaustive discussion, my offering is intended to be suggestive of the sorts of alternative explanations that I think can be given for many, if not all, of the results that are currently supposed to be evidence the ill-formed negativity bias.

Consider first that many of these results are plausibly explained by the information which the causes transmit to the agent. Discussion of information effects has entered the negativity bias literature by consideration of the *positive-negative asymmetry* hypothesized for impression formation. According to this hypothesis, the first impressions that we form about something are influenced more by the negative traits that we believe it to have than by the positive traits that we believe it to have. The best explanation for the results, however, is a matter of lively debate. In particular, the informational features of the traits—how revealing, extreme, or diagnostic a trait is, for example—are offered as alternative explanations to the positivity or negativity of the trait.^10^ If these explanations are correct, then my belief that you are a liar, for instance, impacts my overall impression of you more than my belief that you are kind because being a liar is a more informative trait than being kind. Or, at least, because I believe that it is a more informative.

In the early days of impression formation research, Kellermann (1984) recognized that informational explanations, which explain first impressions by informational features of traits, are *in competition with* hedonic explanations, which explain first impressions by the valence of traits. Thus she writes (p. 43) that “…it is probably a misnomer to call the [impression formation] effect a negativity effect, it should be called an informativeness effect.” She concludes by discussing how the informativeness explanation, unlike the hedonic explanation, will sometimes predict that a positive trait, e.g., brilliant, will be more influential than a negative trait, e.g., clumsy. We can see that the hedonic explanation for these effects is in competition with informational explanations as these will clash whenever the ‘bad’ (in some fixed sense—which as we’ve seen needs further specification) carries less information than the ‘good’ (in some corresponding fixed sense).

Unfortunately, Kellerman’s understanding that informational explanations are alternative explanations to any specified hedonic explanations has not been widely recognised and has certainly not been recognised as an effective springboard for undermining interpreting these results as supposed support for the hedonic interpretation of them offered in supposed support of the negativity bias. Though initially drawing on the positive-negative asymmetry as evidence, Baumeister et al. (2001), for instance, seem eventually willing to grant that research on impression formation may instead be explained as an informational effect, but they think that the explanatory power of informational features is limited. In discussing Kellermann (1984) they thus write (p.359):

Even if this assessment is correct, however, it is confined to the sphere of forming impressions of newly met acquaintances, so something additional would be needed if there is indeed a more general pattern in which bad is stronger than good.

Notice, however, that informational features are primed to explain much more than just impression formation.

The potent explanatory power of informational features has been acknowledged by most other advocates of the negativity bias who, like Baumeister et al., have nonetheless failed to recognize informational effects as explanatory competitors to the hedonic ones offered in supposed support of the negativity bias. Peeters and Czapinski (1990) and Lewicka et al. (1992), for instance, explain the majority of the data offered in support of the negativity bias as informational effects. They argue that negative stimuli generally have more informational value than positive stimuli. Building on this work, Rozin and Royzman (2001) likewise seem to think that informational asymmetries underpin many of the results they discuss. They write (p. 315):

The demonstrated much lower frequency of negative than positive events, makes the negative events more informative. Hence, this general informational bias would work in the service of the negativity bias.

While these authors recognize the explanatory potency of informational features, they do not consider that the informational features may be in explanatory competition with any hedonic interpretations of the negativity bias which they proceed to offer. Unlike Kellerman, they take these informational effects as *support* for the ill-formed negativity bias. They do not realize that the asymmetry may be informational but *not* hedonic, such that their appeal to informational effects to support acceptance of the negativity bias actually undermines any of the many hedonic disambiguations of it.

As Baumeister et al. and Kellerman seem to recognize: informational and hedonic features are competing explanations for the identified effect in impression formation. As Czapinski, Peeters, Rozin, and Royzman all recognize: the same sorts of informational asymmetries present in impression formation are characteristic of much of the research offered in supposed support of the negativity bias. Putting these together: it is plausible that many of the identified effects which are currently—and wrongly, on the basis of the arguments of the previous section—taken to support the negativity bias are, instead, informational effects. While I lack the space to evaluate all the evidence offered in Baumeister et al. alone, much less the entirety of the corpus, informational effects are, I think, alternative explanations of much of the research wrongly taken as evidence of the negativity bias. Informational effects are worthy of further exploration in their own right.

Related to informational explanations are the underappreciated effects of expectation which may likewise go a long way towards explaining some of the results offered in supposed support the negativity bias. For instance, Baumeister et al. (2001) appeal to multiple studies which they take to evidence that whether a romantic relationship will succeed in the long term is better predicted by the lack of bad interactions than by the presence of good interactions. Expectation seems clearly relevant here. The results may, at least largely, be explained by the fact that the “good” interactions are expected, but the “bad” interactions are not. If my partner insults me, that may make a bigger impression on our relationship than when he gives me a hug—not because an insult is “bad” and a hug is “good” (however we might disambiguate ‘bad’ and ‘good’), but instead because I expect hugs from a romantic partner and I do not expect insults. The ubiquity of unexpected interactions may show that many people’s romantic expectations are unrealistic or irrational, but these expectations are nonetheless plausibly present and causally relevant. Many of the found results—wherein a “bad” interaction seems to have a greater impact on close relationships than a “good” interaction—may be explained by the fact that the “bad” interactions are unexpected, whereas the “good” ones were expected.

The explanatory role of expectation is important for more than just those results concerning close relationships. As noted above, many advocates of the negativity bias take negative events to be rarer than positive events. Insofar as negative events are taken by *subjects* to be rarer than positive events, they may often also be more unexpected, thus paving the way to yet further alternative explanations by expectation of some of the results wrongly supposed to be evidence for the negativity bias. (Wrongly, again, because any supposed evidence could be legitimately taken as confirmation or disconfirmation, given the ill-formation of the hypothesis). So, for instance, subjects may find unexpected events more in need of explanation than expected events, which may explain some subset of the results concerning cognitive elaboration. Subjects may also, for instance, find events that they did not expect to be more memorable. And so on. Many discovered asymmetries across specific measures may be explained by asymmetries in expectation. As with the informational alternatives, it is worth noting that these are explanatory competitors of any specific hedonic features we may be interested in testing: explanation by expectation carries different predictions than explanation by hedonics, in the many cases where these dissociate.

Expectations may also affect hedonic magnitude in underappreciated ways. Schroeder (2004), for instance, argues that expectation is always relevant to the degree of felt pleasantness and unpleasantness. As a result, according to Schroeder, unexpected occurrences pack a greater hedonic punch. Applying this insight to some of the results offered in support of the negativity bias: controlling hedonic magnitude requires controlling expectation. This is rarely done and it is not clear how much asymmetry across many of the specific impacts tested would remain if it were.^11^

An event or experience may not only be unexpected, but momentous, and this plausibly explains yet more of the results. Some types of occurrences are such that a single happening can make a big impact. Indeed, the evolutionary stories offered in explanation of the negativity bias by Baumeister et al. appeal to the fact that many obviously “bad” things—like injury or death—are often momentous. Rozin and Royzman (2001) also, for instance, make much of the supposed fact that the “bad” is often more momentous than the “good.”

Again, however, having rejected the negativity bias, we can see that momentousness may be an alternative explanation—and, indeed, one that will also be a competing explanation for any hedonic ones. So, Baumeister et al. (pp. 327–328) write:

Perhaps the broadest manifestation of the greater power of bad events than good to elicit lasting reactions is contained in the psychology of trauma. The very concept of trauma has proven broadly useful, and many psychologists have found it helpful in many different domains. Many kinds of traumas produce severe and lasting effects on behaviour, but there is no corresponding concept of a positive event that can have similarly strong and lasting effects. In a sense, trauma has no true opposite concept.

This passage is puzzling. If they are correct that traumatic experiences have no opposite, then it is hard to see how it is even a candidate instance of the principle that bad is stronger than good: if the authors are right, we simply have a momentous event with (unsurprisingly) powerful effects. Any asymmetries appear irrelevant and it is the momentousness alone that seems explanatory.

Going further, however, it is not clear why the authors think that traumatic experiences have no clear opposites: epiphanies and revelations at least putatively seem like good candidates. Baumeister et al. acknowledge that (pp. 327–328): “It is possible that such [positive] events have simply eluded psychological study, but it seems more likely that the lack of an opposite concept for trauma indicates the greater power of bad than good single events to affect people.” I am not sure why the authors think this is more likely. They do not say. The unstated reason may be that they generally take what has received most attention so far as a guide to which phenomena are more robust (p.324). Psychologists have had a negative focus such that they may have missed some positive phenomena, they admit, but they think that the negative focus itself indicates that the negative is in fact stronger. The most plausible explanation for this sociological fact, however, is a sociological one.

The purported negative focus of psychological research, however, may in turn explain why many of the tasks demanded of subjects might themselves explain some of the results offered and wrongly supposed to be evidence for the hypothesis. So, for instance, Baumeister et al. appeal to a study in Pennebaker (1993). The study supports that using more negative emotion words when writing about traumatic experiences results in better outcomes than using positive emotion words—in particular better health and academic outcomes. Baumeister et al. write (p.354), “Pennebaker concluded that the participants who consistently expressed the most anxiety, sadness, and other negative feelings were the ones who subsequently showed the greatest gains in health.” The students in this study, however, are writing about traumatic experiences, and surely it is likely that it is because they are dealing with a horrible experience that using negative words proved beneficial. It may, that is, not be using negative as against positive words that is relevant, but the appropriateness of the chosen words for the demanded task. Pennebaker (1993) hints at this, writing (p. 546): “In the short run, confronting upsetting experiences may be psychologically painful and physiologically arousing. In the long run, however, the act of psychologically confronting emotionally upsetting events is associated with improved physical and psychological health.” Negative words may well be more beneficial when confronting negative situations, but I rather doubt that using negative emotion words to describe positive experiences would have the same beneficial effects. Going beyond this particular study, Clore et al. (1994), for instance, review a wide range of results to argue that supposed effects of hedonics on processing are, instead, better predicted by specific processing requirements and task demands. As they write (p. 61):

Affect induced differences in processing strategy have been attributed to differences in attentional resources, differences in the accessibility of procedural knowledge, and differences in motivation, or some combination of these factors. …there are findings that support as well as contradict each of the key proposals [concerning affect-induced differences] and the specific impact of affect cannot be predicted without considering the specific processing requirements presented by the specific task for which mood effects are being examined.

The moral of their story is that (p. 74), “As a result of these contingencies, one cannot expect that particular affective states will have the same type of impact on performance across mood manipulations and tasks.” Neither they nor anyone else, however, seems to have yet recognized that task demands may be an alternative explanation—one which will be a competing explanation for any hedonic disambiguation of the negativity bias. Many offered results continue to fail to disentangle this confounding factor which may be seen to better explain those results upon rejection of the negativity bias.

The moral of this section is that a number of alternative explanations may explain the results offered as supposed evidence for the ill-formed negativity bias. As the negativity bias currently continues to enjoy wide acceptance and avoid critical scrutiny, it is perhaps unsurprising that these explanations have themselves been offered as supposed evidence of the hypothesis that ‘bad is stronger than good.’ As argued in the last section, however, that hypothesis is ill-formed such that we cannot evaluate it: it can neither be confirmed nor disconfirmed by any of these results. As exemplified in this section, those results wrongly supposed to be evidence for the negativity bias may nonetheless be explained by information features, expectation, momentousness, sociology, task demands, and various combinations of these. This list is not exhaustive, nor is my discussion of those results currently or potentially supposed to be evidence. I nonetheless hope that I have done enough to show that many of those results are subject to widely accepted explanations that remain after the negativity bias is rejected—explanations which, to note a final time, are *competing* explanations for any of the many possible hedonic disambiguations of the negativity bias, discussed further below. The negativity bias ought to be rejected, but many plausible alternative explanations of subsets of the results offered as supposed evidence for it remain worthy of further investigation.

## Conclusion: Alternative Hedonic Hypotheses

I have argued that the negativity bias is unclear in ways that fatally problematize its evaluation and that the results offered for the ill-formed hypothesis are subject to a number of alternative explanations that deserve to be tested and developed in their own right. Even as I think these alternative explanations should be investigated, in concluding, I acknowledge that a plethora of specifications of the negativity bias remain despite the arguments offered above..^12^

While I’ve argued that the wide-sweeping generalization that bad is stronger than good should be rejected, we might consider something to be a specified version of the negativity bias if it further specifies ‘bad’, ‘stronger’, ‘good’, and the intended subjects of these predicates. Which, if any, specified versions of the principle holds will determine which, if any, theoretical and practical morals are implied. Though I leave the implications of each as an exercise for the reader, I briefly conclude by identifying some such alternative hedonic hypotheses.

At the end of the section 2, I distinguished the following two claims:

1. Bad psychological states are stronger than good psychological states.
2. Bad inputs to psychological states are stronger than good inputs to psychological states.

Consider now the following further specifications of claim 1:

- 1A: States with negative hedonic tone consume more attention than states with positive hedonic tone.
- 1B: States with negative hedonic tone take longer to process than states with positive hedonic tone.
- 1C: States with negative hedonic tone facilitate learning more effectively than states with positive hedonic tone.

Similarly, consider these disambiguations of claim 2:

- 2A. Events that an agent judges to be negative cause more cognitive processing than events that an agent judges to be positive.
- 2B. Events that an agent judges to be negative are better remembered than events that an agent judges to be positive.
- 2C. Events that an agent judges to be negative are learned about more easily than events that an agent judges to be positive.

And note that these are distinct from the following:

- 2A*Events that are harmful to an agent cause more cognitive processing than events that are beneficial to an agent.
- 2B* Events that are harmful to an agent are better remembered than events that are beneficial to an agent.
- 2C*. Events that are harmful to an agent are learned about more easily than events that are beneficial to an agent.

Any or all of these alternative hedonic hypotheses may fail to be fruitful and will often compete with those alternatives offered in section 3. As repeatedly noted in that section, for many of the results currently supposed to be evidence of the negativity bias in its current ill form, any hedonic specifications of the kind above will be explanations that compete with those there discussed, i.e. explanation through information, expectation, and so on will compete with all of 1A-2C above for reasons discussed in the previous section. Such specified versions of the negativity bias will also still be difficult to evaluate. Difficulties arising from hedonic complexity remain and will need to be addressed. Evaluating 2A*-2C*, for instance, requires grappling with the fact that an event may be both harmful in some ways and beneficial in others. Difficulties arising from hedonic magnitude likewise remain and will need to be addressed. Evaluating 1A-1C, for instance, requires identifying and controlling the overall degree of positive and negative hedonic tone of the states being compared. This is, of course, not to say that such difficulties could not *be* addressed. Nonetheless, I suggest that if many of the results that led us to the negativity bias are better explained non-hedonically, then we may do best to pursue these alternative and competing non-hedonic explanations before considering which, if any, evaluable specifications of the negativity bias are salvageable.

I will not and cannot here evaluate these distinct hypotheses. Instead, I will grant that though the negativity bias is fatally problematic there are nonetheless plausible alternative explanations for many of the results offered as supposed evidence for the negativity bias that remain worthy of investigation (section 3), and a plethora of specified versions of the hypothesis that may yet prove fruitful (as exemplified above). The negativity bias—despite its wide acceptance in affective science and recent affirming introduction into philosophy—is nonetheless ill-formed. The broad claim that bad is stronger than good should be rejected.

In closing, consider the following plausible cases:

- In a single day, you receive news concerning recent auditions for two plays in which you are equally interested, with directors of comparable prestige. The day is a happy one: the elation you feel from the acceptance overwhelms the deflation you feel from the rejection.
- During an evening at the pub, you are introduced to a friend of a friend who is awkward, shy, and seemingly dull. Left alone at the table, you are forced to strike up a conversation and discover that your new acquaintance has little interest in philosophy or psychology, is a bit short-tempered, and is a member of a different political party. After a pint, just as you are about to disentangle yourself, they mention your favourite book. You begin the conversation with renewed vigour and spend the rest of an enjoyable evening discussing art and literature.
- You are having a lousy, rainy day of work while your beloved is out of town. Hurrying along to grab a sandwich, you encounter a new colleague you don’t yet well know. They approach you with a smile, offering a compliment about your recent presentation, before going on their way. The interaction stays with you and, despite an afternoon involving many of your least favourite administrative tasks, you find yourself humming.

We should not conclude from the wide range of everyday cases that we might similarly describe that ‘good is stronger than bad.’ We have to do the hard work of teasing apart the many specifications of ‘good’ ‘stronger’ and ‘bad’ in order to identify the many, often competing, well-formed hypotheses which may be tested. And then we must test them.

It is time to rethink the idea that ‘bad is stronger than good’—however well-accepted that ill-formed hypothesis may be.

## Footnotes

1 This is not to deny that related hypotheses have occasionally made appearances in philosophical discussions, particularly in ethics and value theory. See, for instance, Hurka (2010) and references therein.

2 Rozin and Royzman’s (2001) is regularly cited and Baumeister et al. (2001) and Rozin and Royzman (2001) cite each other as having independently alighted on the same hypothesis. As discussed in text below, variations on the criticisms offered here are likewise applicable to Rozin and Royzman (2001).

3 All references to Baumeister et al. are to their (2001).

4 Wide acceptance is, of course, not universal acceptance. I am sure there are many working in affective science that do not recognize the negativity bias.

5 Crucially for the applicability of the offered critique, note that we are focused here only on the measures of strength offered in this landmark paper—were we to consider the measures of strength from across the wide literature, we would see that the notion is yet more stretched. The following critique, concerning ‘strength’, is in this case even worse when we apply it beyond Baumeister et al.

6 And again, the problem only intensifies when we move beyond them.

7 As any hedonistic utilitarian will know. For some valiant attempts see Hurka (2010) and Massin (2014).

8 As insightfully discussed by Massin (2014), hedonic scaling may also vary across domains. Distinct types of experience may involve hedonic dimensions with distinct scalar properties, e.g., distinct midpoints and distinct functions holding across the points on the scale. If so, then identifying equivalent hedonic magnitudes will be yet more difficult.

9 There is a further complication if we note that we may think it psychologically good when there is a hedonic match and psychologically bad when there is a hedonic mismatch. For instance, we may think that it is a psychological good to have a negative reaction in response to a negative event, and a positive reaction in response to a positive event. None of the examples in the literature on the negativity bias seem to be of this sort, however, so I set this kind of psychological good aside.

10 I set aside the differences among informational hypotheses. For discussion see Skowronski and Carlston (1989).

11 Bohner et al. (1988) may be thought an exception: they take themselves to find an asymmetrical effect for valence that is unexplained by expectation. I am sceptical, however, that valence and expectation are successfully separated in this study. Expectation was measured by self-reports given after subjects were offered population statistics, and it seems to me that subjects would have therefore been biased to respond in accordance with those statistics—as is consistent with the found results. If you tell me that 90% of people failed a given test, I am less likely to report myself as expecting to pass, even if that is what I *do* expect. Regardless, replication is needed—both in this particular case and across other domains. I know of no more recent even seeming exceptions in the literature.

12 As noted in text below, whether any of these specifications are fruitful goes beyond what I can here settle.
